# Immunoinformatic Analysis of SARS-CoV-2 Nucleocapsid Protein and Identification of COVID-19 Vaccine Targets

**DOI:** 10.3389/fimmu.2020.587615

**Published:** 2020-10-28

**Authors:** Sergio C. Oliveira, Mariana T. Q. de Magalhães, E. Jane Homan

**Affiliations:** ^1^ Departamento de Bioquímica e Imunologia, Instituto de Ciências Biológicas, Universidade Federal de Minas Gerais, Belo Horizonte, Brazil; ^2^ Instituto Nacional de Ciência e Tecnologia em Doenças Tropicais (INCT-DT), Conselho Nacional de Desenvolvimento Cientifico e Tecnologico (CNPq), Ministerio de Ciencia e Tecnologia (MCT), Salvador, Brazil; ^3^ ioGenetics LLC, Madison, WI, United States

**Keywords:** severe acute respiratory syndrome coronavirus 2, Coronavirus Disease 2019, epitopes, vaccine, T cells, B cells, nucleocapsid

## Abstract

COVID-19 is a worldwide emergency; therefore, there is a critical need for foundational knowledge about B and T cell responses to SARS-CoV-2 essential for vaccine development. However, little information is available defining which determinants of SARS-CoV-2 other than the spike glycoprotein are recognized by the host immune system. In this study, we focus on the SARS-CoV-2 nucleocapsid protein as a suitable candidate target for vaccine formulations. Major B and T cell epitopes of the SARS-CoV-2 N protein are predicted and resulting sequences compared with the homolog immunological domains of other coronaviruses that infect human beings. The most dominant of B cell epitope is located between 176–206 amino acids in the SRGGSQASSRSSSRSRNSSRNSTPGSSRGTS sequence. Further, we identify sequences which are predicted to bind multiple common MHC I and MHC II alleles. Most notably there is a region of potential T cell cross-reactivity within the SARS-CoV-2 N protein position 102–110 amino acids that traverses multiple human alpha and betacoronaviruses. Vaccination strategies designed to target these conserved epitope regions could generate immune responses that are cross-reactive across human coronaviruses, with potential to protect or modulate disease. Finally, these predictions can facilitate effective vaccine design against this high priority virus.

## Introduction

The pandemic Coronavirus Disease 2019 (COVID-19) is a worldwide threat caused by the severe acute respiratory syndrome coronavirus 2 (SARS-CoV-2) (1). By July 2020, SARS-CoV-2 had infected over 16 million people worldwide and killed more than 645,000 individuals. A better understanding of the immunogenicity and pathogenesis of SARS-CoV-2 infections in humans is thus urgently needed as a basis for the development of new vaccines against SARS-CoV-2 (2).

The coronaviral genome encodes a relatively small number of proteins, classified as either structural or non-structural. Among structural proteins, the spike glycoprotein (S), and the nucleocapsid protein (N) are the major ones, while the envelope protein (E) and membrane protein (M) are smaller structural components (3, 4). The spike (S) protein is arrayed on the surface of the virus particles, giving the characteristic ‘crown’ appearance (5). The S protein comprises two subunits: S1 and S2. The S1 subunit consists of an amino-terminal domain and a receptor-binding domain (RBD) (5, 6). The RBD binds to ACE2 as its host cell target receptor, which allows virus entry (5, 7). Various reports related to SARS-CoV-2 suggest a correlation between neutralizing antibodies and the number of specific T cells to viral particles (8). Some vaccine candidates have been shown to protect from infection in laboratory animals models (9). Most vaccine studies so far have focused on antibody responses generated against the S protein, the most exposed protein of SARS-CoV-2 (10, 11). However, antibody responses are not detectable in all infected patients, especially those with less severe forms of COVID-19 (12). Previous studies with SARS-CoV-1 have also shown that memory B cell responses tend to be short-lived after infection (13). In contrast, memory T cell responses can persist for many years (14), and in mice, these protect against lethal challenge with SARS-CoV-1 (13). Additionally, the spike protein has several hotspots for mutations (15), whereas the nucleocapsid gene is more stable and has acquired fewer mutations to date (16).

In this study, we focus on the SARS-CoV-2 nucleocapsid protein that is involved in viral pathogenesis (4, 17). The nucleocapsid is the most abundant protein in coronaviruses, is highly immunogenic, and its amino acid sequence is largely conserved as previously reported (4). Therefore, this protein has advantages as a candidate for vaccine development (4, 18). Previous studies on SARS-CoV-1 reported N protein epitopes as capable of eliciting massive production of antibodies in infected subjects (4). T cell responses to SARS-CoV-1 are in some cases shown to last up to 11 years thus representing a valid alternative for the design of vaccines (4, 19). Monkeys vaccinated with an adenovirus vectored SARS-CoV-1 vaccine were shown to have consistent T cell responses to the N protein (20). Similarly in MERS the nucleocapsid has been examined as a potential vaccine candidate (21, 22). Recall responses of T cells reacting with peptides of SARS-COV-2 N protein have been demonstrated in both SARS-CoV-1 recovered patients, 17 years after exposure, and those with no history of SARS-CoV-1 exposure (23, 24). Preliminary studies of SARS-CoV-2 have also demonstrated antibodies directed to the N protein (2).

Studies involving computer simulations for the identification of the epitopes recognized by antibodies and T cells are central to immunological applications such as drug design and vaccine development. Bioinformatics tools offer the advantage, in addition to speed and biosafety, of being unbiased by peptide selection. Approaches which use overlapping peptides, spaced other than single amino acid displacement, may exclude the key peptides. There have been several reports of bioinformatics analyses of SARS-CoV-2 using a variety of platforms (25–29). Herein, we applied bioinformatics analysis to determine the antigenic potential of the SARS-CoV-2 N protein. Major B and T cell epitopes of the SARS-CoV-2 N protein are predicted and these peptides were compared to other coronaviruses that infect humans. As other studies have suggested that prior exposure to less virulent human coronaviruses may confer some protection (24, 30–32), we focused particularly on identifying conserved motifs which potentially could elicit cross-reacting T cell responses through shared T cell exposed peptides. The epitope mapping and comparison of potential cross-reactive epitopes presented in this study may provide an opportunity for the development of new vaccines and immunodiagnostic tools. Finally, the sudden emergence of SARS-CoV-2 apparently from bats is an indicator that similar betacoronaviruses could emerge in the future. It is therefore of interest to determine if there are potential antigens that are conserved and could cross protect against future zoonotic coronaviruses.

## Material and Methods

### Accession Numbers

Accession numbers of the nucleocapsid proteins analyzed are as follows: HKU1:YP_173242.1; 229E:NP_073556.1; MERS: YP_009047211.1; NL63:YP_003771.1; OC43: YP_009555245.1; SARS COV1:NP_828858.1; SARS-COV2: YP_009724397.2.

### Determination of Predicted Epitopes for SARS-CoV-2 Nucleocapsid

B cell linear epitope probability and MHC binding affinity were determined for all sequential peptides with a single amino acid displacement, using an updated version of methods previously described (33, 34). Briefly, in lieu of representing peptides as simple alphabetic sequences, multiple physicochemical properties of each amino acid are transformed to mathematical vectors by principal component analysis. Using a training set of known MHC binding reactions, B cell epitope binding and cathepsin cleavage reactions, neural networks are used to derive predictive equations applicable to any peptide. Predictions are made for 70 MHC I alleles and 65 MHC II alleles. To estimate population behavior comprising multiple MHC alleles with varying affinities for any peptide, the LN ic50 binding data estimates were transformed and standardized to a zero mean unit variance within each protein using a Johnson Sb distribution (35). To compute a permuted average across human alleles, the highest predicted binding affinity at each peptide position was determined for every possible haplotype pairing and averaged; this was computed using predicted binding for 31 MHC IA, 31 MHC IB, and 24 DRB alleles as previously demonstrated (36). Predictions of the probability of cathepsin cleavage at each dimer were similarly derived by training on known cleavage reactions (34). These predictive methods have been experimentally validated in proteins of multiple origins (34, 37–40).

### Nucleocapsid Sequence Alignments and Structural Analysis

Several protein sequences were analyzed by using the Basic Local Alignment Search Tool specific for protein sequences (BLASTp) (41). Multiple sequence alignments were prepared with Clustal Omega (multiple sequence alignment) and manually edited in *py*BoxShade 3.21 (https://github.com/mdbaron42/pyBoxshade). We selected statistically significant matches to calculate a similarity tree for related coronaviruses. The epitopes were mapped based on the amino acid physical–chemical properties and location at possible areas of cross-reactivity and antigen-binding by using an in-house software (data not shown). Analysis of the protein secondary structure prediction and annotation was carried out with PSIPRED Protein Analysis Workbench (http://bioinf.cs.ucl.ac.uk/psipred/) (42, 43). The epitopes were identified, built in Chimera v.1.13.1. We also used Chimera to prepare images and calculate RMSD between sequences (44). Distance between residues were measured by using wizard measurement tool from PyMOL (The PyMOL Molecular Graphics System, Version 1.2r3pre, Schrödinger, LLC.).

### T Cell Exposed Motifs

All sequential T cell exposed motif patterns were extracted from each protein and ranked as previously described for each of three recognition patterns of amino acids which engage T cell receptors (33, 36, 45). These T cell exposed recognition patterns comprise the amino acids not hidden in pocket positions. These are positions ~~~4,5,6,7,8~ within a MHC I binding 9-mer and ~2,3,~5~7,8~ or −1~~,3,~5~7,8~ relative to the 9-mer core of a MHC II binding 15-mer.

## Results

### SARS-CoV-2 Nucleocapsid B and T Cell Epitope Mapping

**Figure 1:**
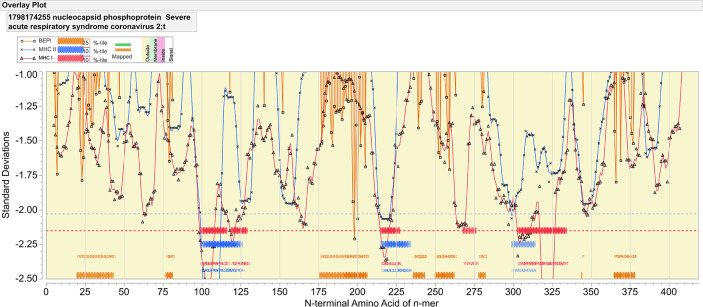
Epitope mapping of nucleocapsid protein of SARS-CoV-2. The X axis indicates the index position of sequential peptides with single amino acid displacement. The Y axis indicates predicted binding affinity in standard deviation units for the protein. The red line shows the permuted average predicted MHC-IA and B (62 alleles) binding affinity by index position of sequential 9-mer peptides with single amino acid displacement. The blue line shows the permuted average predicted MHC-II DRB allele (24 most common human alleles) binding affinity of sequential 15-mer peptides. Orange lines show the predicted probability of B-cell receptor binding for an amino acid centered in each sequential 9-mer peptide. Low numbers for MHC data represent high binding affinity, whereas low numbers equate to high B cell receptor contact probability. Ribbons (red: MHC-I, blue: MHC-II) indicate the 10% highest predicted MHC affinity binding. Orange ribbons indicate the top 25% predicted probability B-cell binding. Horizontal dotted lines demarcate the top 5% of binding affinity for the protein (red MHC I, blue MHC II).

The nucleocapsid of SARS-Cov-2 exhibits both strong B and T cell epitopes distributed across the whole protein. **Figure 1** provides an overview map of both probable linear B cell epitopes and regions of predicted high affinity MHC binding for multiple alleles. Corresponding sequences of predicted antigenicity are shown in **Table 1**. As shown in **Figure 1**, we predicted multiple high probability B cell linear epitopes. A 9-mer peptide was scored as “high probability” if they were predicted to be in the top 25% of probability of being in a B cell epitope for the protein as a whole. The most dominant of these lies between 176 and 206 in the sequence SRGGSQASSRSSSRSRNSSRNSTPGSSRGTS. Additional high probability B cell epitopes are indicated in **Table 1**. When analyzed by the same immunoinformatic approach alongside all structural proteins in the virion, the nucleocapsid B cell epitope at 176–206 stands out as dominant with respect to the epitopes in the spike glycoprotein (data not shown).

**Table 1:** SARS-CoV-2 predicted antigenicity of B and T cell epitopes.

B cell epitopes	
**Position**	**Peptide sequence**
21–32	SDSTGSNQNGER
76–82	TNSSPDD
176–206	SRGGSQASSRSSSRSRNSSRNSTPGSSRGTS
235–243	SGKGQQQQG
249–263	KSAAEASKKPRQKRT
363–379	FPPTEPKKDKKKKADET
**MHC I binding regions for multiple alleles**	
**Position**	**Peptide sequence**
97–137	GDGKMKDLSPRWYFYYLGTGPEAGLPYGANKDGIIWVATEG
209–232	RMAGNGGDAALALLLLDRLNQLES
261–279	KRTATKAYNVTQAFGRRGP
306–335	QFAPSASAFFGMSRIGMEVTPSGTWLTYTG
**MHC II binding regions for multiple alleles**	
**Position**	**Peptide sequence**
97–127	GGDGKMKDLSPRWYFYYLGTGPEAGLPYGANK
213–238	NGGDAALALLLLDRLNQLESKMSGKG
293–320	RQGTDYKHWPQIAQFAPSASAFFGMSRI

**Figure 2:**
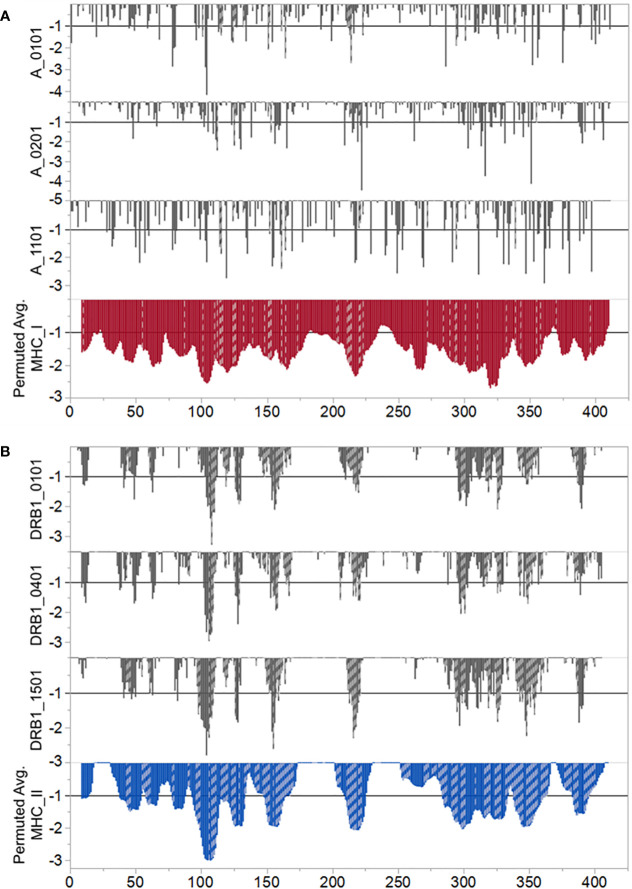
Predicted differential binding of example alleles. **(A)** MHC I and **(B)** MHC II. In both panels the Y axis indicates predicted binding affinity of sequential peptides. The X axis indicates the index position of each 9 mer (MHC (I) or 15-mer (MHC II) represented by a vertical bar. Bars which are cross hatched are those peptides predicted to be excised for binding and presentation by either cathepsin S or cathepsin L. For MHC I the cathepsin predictions are those which excise a 9 mer. For MHC II a predicted excision of a 12–18 mer is shown. The lower tier of each panel shows the population permuted average predicted binding affinity as described for **Figure 1**. The top three tiers contrast the responses of selected example alleles. For MHC I we show predicted responses of A_0101, A0201, and A1101. For MHC II we show predicted responses of DRB1_0101, DRB1_0401, and DRB1_1501. Other alleles evaluated show a similar diversity of predicted response.

**Figure 1**, in which consideration is given to the predicted binding of multiple common human MHC I and MHC II alleles, indicates three regions of predicted high MHC II binding and four regions of high affinity MHC I binding for multiple alleles, which comprise the top 10% highest predicted affinity for the protein. These are shown in **Table 1**. However, as the examples shown in **Figure 2** underscore, there are differences in MHC allele-specific binding. The differences are more marked for MHC I, where binding is often restricted to one or two sequential 9-mers, whereas the broader sequences identified for MHC II, tend to span more alleles. For example, adjacent to the dominant B cell epitope we see that a DRB1_1501 has a stronger predicted MHC II binding which could indicate more T cell help than is the case for an individual of DRB1_0101. Furthermore, when consideration is given to probable cathepsin cleavage, not all peptides may actually be presented. However, we appreciate that cathepsins play a major role in generating peptides to be presented for the vacuolar pathway (endolysosomes and phagosomes) as demonstrate by Shen et al. (46). Therefore, cathepsins are primarily involved in TAP-independent MHC class I crosspresentation. Nevertheless, this analysis suggests that individuals of different immunogenetics would be expected to show differing responses. The proximity of MHC binding sequences to the B cell epitopes at 76–82 and 176–206 amino acids indicates these epitopes may also receive strong epitope specific T cell help.

### Conservation of T Cell Epitopes Among Coronaviruses

**Figure 3:**
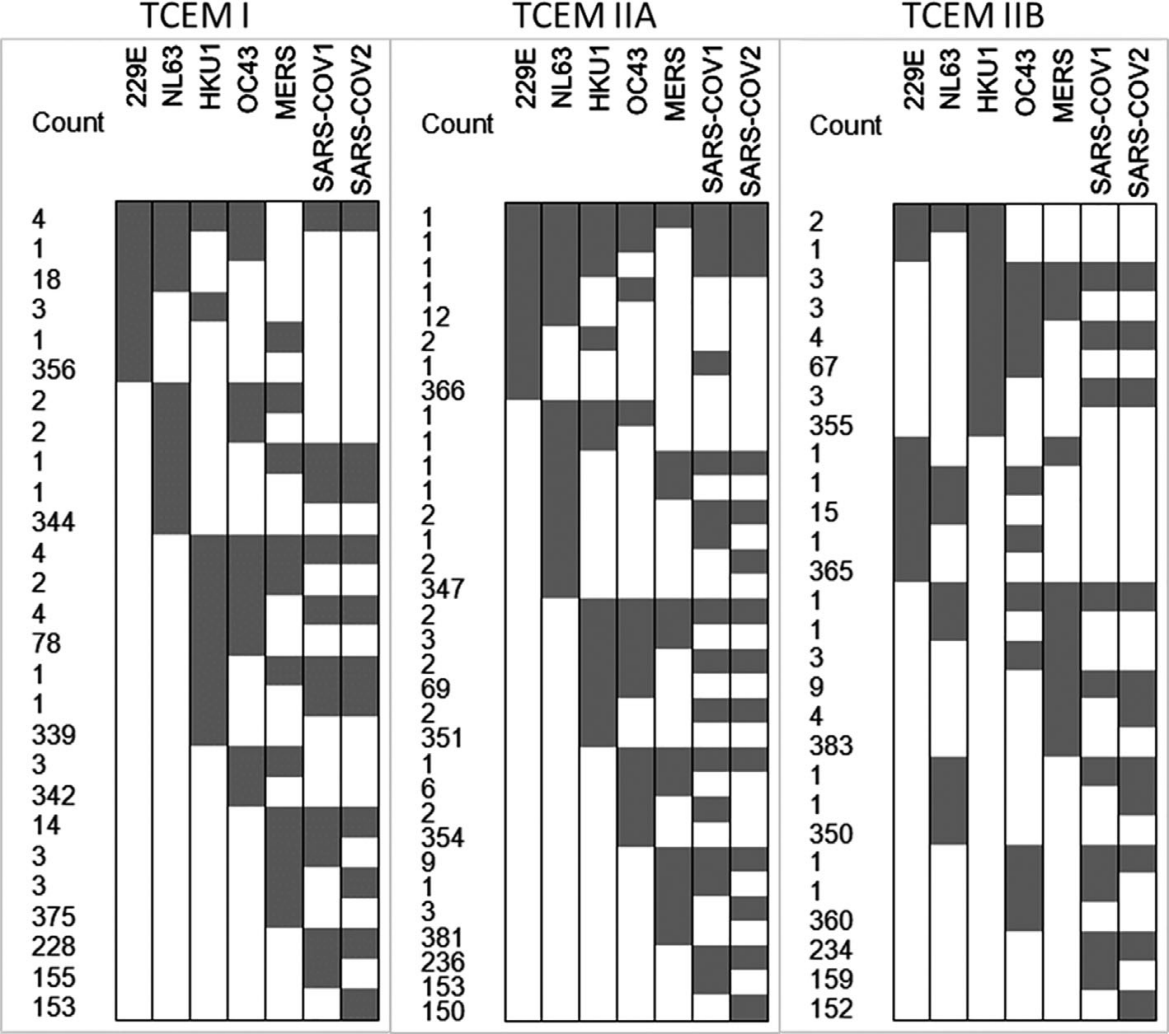
T cell exposed motifs conserved across coronaviruses. The cell plots show in gray where there are T cell exposed motifs shared between SARS-CoV-2 and other human coronaviruses, as shown in the X axis. The number of shared motifs indicated in the Y axis counts. The most highly conserved motifs are also shown in **Supplementary Table 1**.

**Figure 4:**
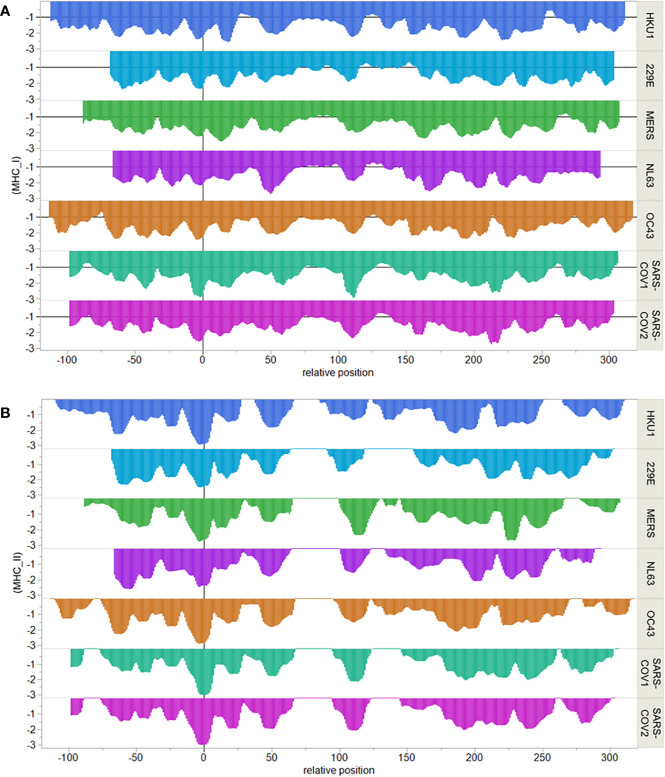
Comparative MHC binding patterns in human coronavirus nucleocapsid protein. X axis shows sequential peptides aligned relative to the most conserved MHC I pentamer. Peptides are 9-mers for MHC I and 15-mers for MHC II and are indicated at their index positions. Y axis shows permuted predicted binding in standard deviation units below the mean for the protein. **(A)** shows MHC I alleles. **(B)** shows MHC II DRB alleles.

We next compared the epitope map of SARS-COV-2 N protein to that of other coronaviruses known to have infected humans. Here, we focused on the T cell exposed motifs, which indicate where potential T cell cross-reactivity may occur. A single T-cell receptor engages only with the few amino acids of a bound peptide MHC that are protruding from a MHC histotope, together with contact points within the histotope. We refer to this pentamer motif as the T cell exposed motif (36). **Figure 3** shows the patterns of T cell exposed motif sharing between human alphacoronaviruses 229E and NL63 with betacoronaviruses HKU1, OC43, MERS, SARS-CoV-1, and SARS-CoV-2. While some of the T cell exposed motifs are conserved, the flanking regions of these peptides, comprising the groove exposed motif, differ. Most notably there is a region of potential T cell cross-reactivity within the SARS-CoV-2 N protein position 102–110 that traverses the human alpha and beta coronaviruses, except for MERS. In MERS substitution of Leu>Thr at the SARS-CoV-2 position 113 (equivalent to the MERS 103 position) removes the conservation of the T cell exposed motifs with SARS-CoV-2. The region in which the conserved motifs occur is also predicted to have high affinity binding for multiple MHC I and II alleles. Here, we used 70 human MHC I and 65 MHC II alleles for our analysis of permuted binding that represents about 85% of human population. The T cell motif sharing is further extended within the betacoronaviruses. The conserved T cell exposed motifs are shown in **Supplementary Table 1**. When the N proteins of the six viruses sharing most motifs are aligned at the peptide comprising the most conserved T cell exposed MHC I motif ~~~FYYLG~ (in SARS-CoV-2 position 107), the commonality of epitope patterns is evident (**Figure 4**).

### 3-D Structure Model of Nucleocapsid From Different Coronaviruses

**Figure 5:**
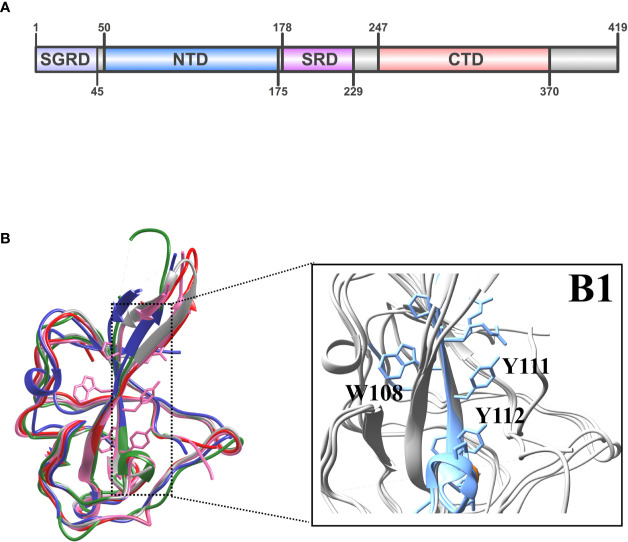
**(A)** Domain organization of coronaviral N proteins. The four domains labeled are as follows: SGRD, serine–glycine–arginine-rich domain; NTD, N-terminal domain; SRD, serine-rich domain; and CTD, C-terminal domain. **(B)** Superimposition of the HCoV-Sars-2 NTD in pink (pdb ID: 6M3M) with NTDs from Sars-CoV-1 in red (pdb ID: 2OFZ), HCoV-OC43 in green (pdb ID: 4J3K), HCoV-NL63 in blue (pdb ID: 5NK4), MERS in gray (pdb ID: 4UDI). (B1) The beta-strand (*β*3) region for the major conserved epitope is highlighted in blue with the two conserved tyrosines for RNA binding.

The coronavirus nucleocapsid protein consists of two folded domains (NTD and CTD) linked by an unstructured region (47). In more details the N protein includes the following domains: serine–glycine–arginine-rich domain (SGRD), N-terminal domain (NTD), serine-rich domain (SRD), C-terminal domain (CTD) as described in **Figure 5A** (48, 49). Our alignment has revealed that despite the conservation of some motifs, the N protein from various different coronaviruses often exhibit different properties, due primarily to their otherwise low sequence homology (_˜_50%) (**Supplementary Figure 1**). The structural similarity appears to be at the whole folded level with its five-stranded anti-parallel *β*-sheet sandwiched between loops (or short 3–10 helix) on the outside (**Figure 5B**). Several nucleocapsid NTD domains are similar in topology and surface electrostatic profiles as observed. The root mean square deviation (RMSD) between the structures coordinates is 0.867 Å over superimposed C atoms. The most dramatic differences can be observed in loops L1 (between *β*2 and *β*3, residues 96 to 104) and L3 (residues 119 to 128). Other authors also observed that strands *β*2 and *β*3 are connected by a long flexible loop composed of amino acid residues 96 to 104 protruding out of the core (50, 51). We could identify and observe (**Figure 5B**) the structure of the highly conserved twelve-residue peptide corresponding to the region _107_RWYFYYLGTGPY_118_ (YP_009724397.2). This peptide is located at the NTD of N protein, close to the L1 loop and has a conserved and important epitope located in an exposed beta-strand, with two exposed tyrosines (**Figure 4B1**). Both tyrosines (Y111 and Y112) have been proposed to be involved in RNA recognition, stacking with consecutive nucleotide bases. The NTD of the N protein from the selected coronavirus was compared to assess the similarity level existing between the conserved protein sequences of the human coronaviruses (4). Structural mapping of the epitopes shown in **Figure 5B1** into 3D models of the NTD N protein (6M3M, 5NK4, 4J3K, 1SSK, 4UD1 entries of PDB database) reveals a conserved epitope predicted in a highly immunogenic peptide exposed to the extracellular environment, likely, to other host immune system components. We were also able to demonstrate that the predicted B cell epitope in SARS-CoV-2 at _176_SRGGSQASSRSSSRSRNSSRNSTPGSSRGTS_206_ is inside the unstructured region inside of SGRD domain of SARS-CoV-2 (**Supplementary Figure 1**, sequence colored in blue). Unfortunately, this region could not be mapped in the 3D model due to the lack of a structure model for the whole protein length.

## Discussion

COVID-19 pandemic challenged the world to speed up research for a vaccine against SARS-CoV-2 infection. Despite massive effort and many thousands of studies published within the first 8 months of the pandemic, our understanding of how humans respond to SARS-CoV-2 is still quite limited (2). Worldwide efforts are currently underway to map the determinants of immune protection against SARS-CoV-2. In this study, we used a bioinformatics approach to map B and T cells epitopes in the nucleocapsid protein of SARS-CoV-2. The SARS-CoV-2 S protein is being studied as the leading target antigen in vaccine development (52, 53). However, a better understanding of viral entry is required to avoid further complications with the vaccine immune response, similar to those observed with HIV type 1 (HIV-1) Env protein candidate vaccine (53, 54). Additionally, the spike protein has several hotspots for mutations (15). In contrast, the nucleocapsid gene is more conserved and stable, with fewer mutations over time (16). Nucleocapsid proteins of many coronaviruses are highly immunogenic and are expressed abundantly during infection (53, 55). High levels of IgG antibodies against nucleocapsid have been detected in sera from SARS patients (53, 56), and the N protein is a representative antigen for the T-cell response in a vaccine setting (20, 53).

In this study, our bioinformatics analysis was able to identify epitopes conserved in several human coronavirus N proteins. The results show that there are several overlapping conserved peptides. When combined, our analysis could thus predict not only high binding individual 9-mer peptides, but also highly exposed structural regions of immunological peptides, which could have potential importance as candidates for vaccines. Our findings are consistent with the strong antigenicity previously noted in SARS N protein and prior reports for SARS-CoV-2 (24). The predicted B cell epitopes we identify are consistent with the strong IgG, IgM, and IgA responses to the N protein in an acutely infected patient documented by Dahlke et al. using peptide arrays (2) and with the observations of Grifoni et al. (31). We identified a strong immunodominant B cell epitope SRGGSQASSRSSSRSRNSSRNSTPGSSRGTS between 176 and 206 amino acids in the nucleocapsid protein sequence. With appropriate T cell help this epitope may be a good target for neutralizing antibodies and long-lived immune response.

Additionally, we performed an *in-silico* survey of the major T cell epitope sequences of the nucleocapsid protein from coronaviruses known to have infected humans (4). The demonstration of conserved T cell exposed motifs between the N protein of multiple human coronaviruses may account for the reported recall of T cell responses over decades, even in the absence of SARS-CoV-1 exposure (23, 57). We found a region of potential T cell cross-reactivity within the SARS-CoV-2 N protein positions 102–110 and equivalent positions in the human alpha and beta coronaviruses, with the exception of MERS. Comparison of the individual allele predicted binding affinities to the SARS-CoV-2 peptides shows differences in responses based on individual genetics. The conserved T cell exposed motifs shared between coronaviruses are each contextualized in different flanking regions comprising pocket positions that will bind with differing affinities. These complexities underscore the nuanced differences in individual patient’s responses. As much of the pathogenesis of COVID-19 disease appears linked to the immune and inflammatory response, it is important to keep in mind that individual differences in clinical response may be rooted in the patients MHC alleles as well as in presence of the preexisting cognate T cell clones, which may have been primed by different peptides. We also address the potential T cell epitopes by a complementary structural bioinformatics method, which was able to assess the conservation of these epitopes across different human coronaviruses. We explored the fact that 89.74% of amino acid sequence of the N protein of SARS-CoV-1 is similar to SARS-CoV-2, with high similar 3D structures demonstrated by homology modeling, and biophysical feature comparison (58). The relevant amino acids are close to a highly dynamic loop, which is important for the protein primary biological function as the scaffolding agent for the viral genomic stability (59).

The role and diversity of the T cell response to SARS-CoV-2 was reviewed by Altmann and Boyton (60). There have been multiple efforts to map epitopes in the viral proteome, using both bioinformatics and *ex vivo* approaches. While most of these have prioritized the spike protein, several epitopes in the N protein have been reported. Mateus et al. identify CD4+ T cell allele-specific epitopes encompassed in the sequences we identify from positions 213–238 to 293–320 as binding multiple MHC II alleles (30). Most notably, our findings parallel those of Le Bert et al. (24) who demonstrated CD4+ and CD8+ T cell responses to peptides that overlap the multiallelic binding regions we predicted. In particular, patients who were not exposed to SARS-CoV-2 had CD4+ T cells responsive to N101–120, which comprises the most conserved T cell exposed motifs (**Supplementary Table 1**).

The existence of broadly conserved T cell exposed motifs in the N protein indicates that, even while peptide context is different, there may be potential to develop a vaccine which offers protection across multiple coronaviruses. This was addressed for MERS by Shi et al. (21). Among the epitopes they identified, there are several CD8+ T cell peptides in homologous positions to those we have predicted in SARS-CoV-2, although as noted the T cell exposed motifs conserved in SARS, SARS-CoV-2 and the other human coronaviruses do differ from MERS. Yang et al. also proposed a nucleocapsid based vaccine for SARS-CoV-1 (61).

In summary, the use of available information related to SARS-CoV-2 epitopes associated with bioinformatics predictions points to specific regions of viral nucleocapsid that are targets to human immune responses (25). We understand that lack of biological confirmation of identified peptides may limit the impact of our discovery. However, testing the antigenicity of these B and T cell epitopes will be the next step on our research program. The observation that some T cell epitopes are highly conserved between SARS-CoV-2 and other human coronaviruses is critical. Vaccines that target human immune responses toward these conserved epitopes could generate immunity that is cross-protective across alphacoronaviruses and betacoronaviruses (25). This would be an advantage given the potential of future novel coronavirus emergence.

## Data Availability Statement

The raw data supporting the conclusions of this article will be made available by the authors, without undue reservation.

## Author Contributions

Conceptualization: SO, MM, EH. Methodology: SO, MM, EH. Formal analysis: SO, MM, EH. Investigation: SO, MM, EH. Writing: SO, MM, EH. All authors contributed to the article and approved the submitted version.

## Funding

This work was supported by grants from Conselho Nacional de Desenvolvimento Cientifico e Tecnologico (CNPq) grant #465229/2014-0, 401209/2020-2 and 302660/2015-1 (to SO) and Fundação de Amparo à Pesquisa do Estado de São Paulo (FAPESP) grant #2017/24832-6 (to SO) and Coordenação de Aperfeiçoamento de Pessoal de Nível Superior (CAPES) grant #88887.506611/2020-00 and 88887.504420/2020-00 and National Institute of Health (NIH) grant# R01 AI 116453 (to SO).

## Conflict of Interest

EH is an employee and equity holder in ioGenetics LLC.

The remaining authors declare that the research was conducted in the absence of any commercial or financial relationships that could be construed as a potential conflict of interest.
