# Supplementary material for: Immunoinformatic Analysis of SARS-CoV-2 Nucleocapsid Protein and Identification of COVID-19 Vaccine Targets
Source: Front Immunol. 2020 Oct 28;11:587615. doi: 10.3389/fimmu.2020.587615 (PMC7655779; doi:10.3389/fimmu.2020.587615)
Supplement: Supplementary file 1 [file DataSheet_1.pdf]

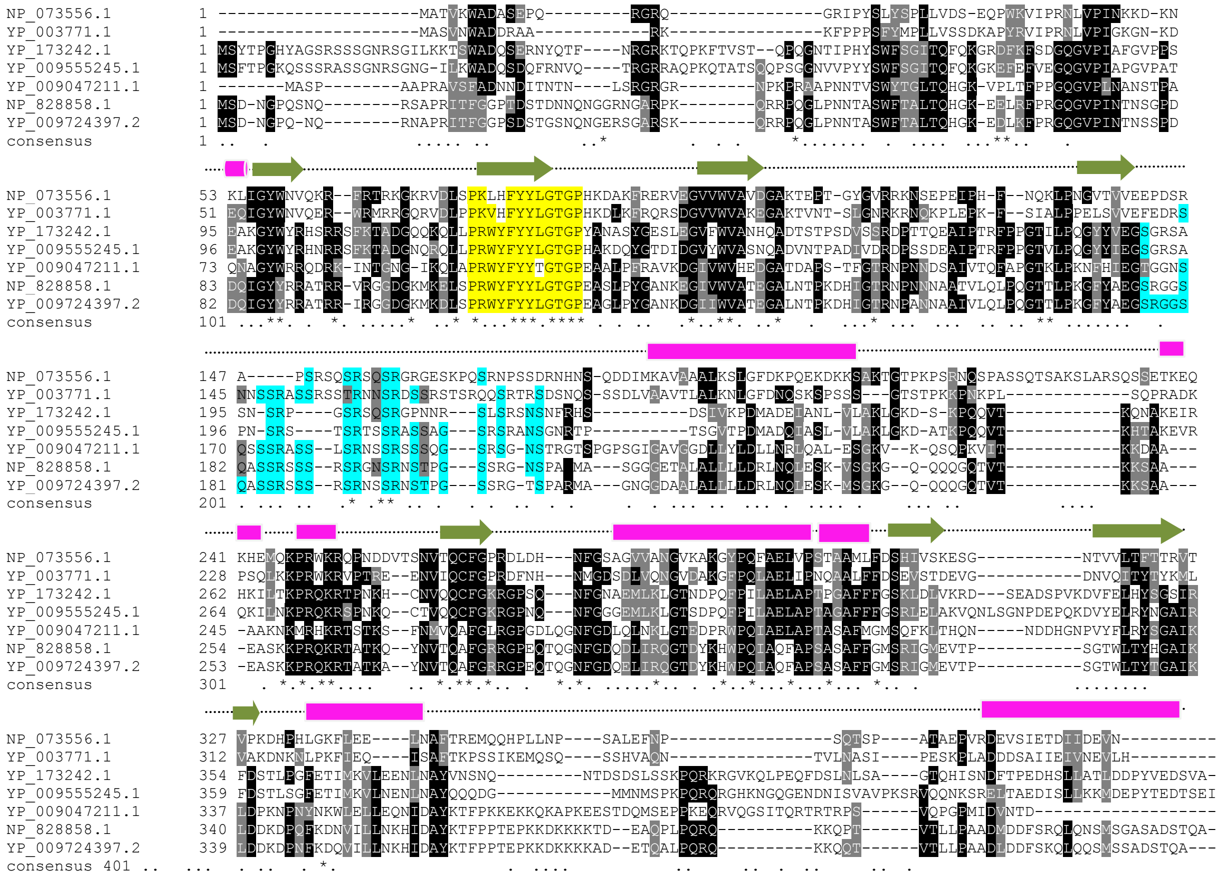

Supplementary Figure 1. Multiple sequence alignment of complete coronaviral N protein. The sequences of N proteins from several coronavirus were aligned by Clustal Omega Server and colored by *py*Boxshade programs. The conserved residues for the important epitopes are shaded in yellow and blue. Completely identical residues are shaded in black and similar residues in gray. The secondary structural alignment elements are draw above the alignment and can be observed in green for beta-strand and pink for alfa-helix. The acronyms used for each viral sequence and their corresponding database accession numbers are as follow: 229E:NP_073556.1; NL63:YP_003771.1;H KU1:YP_173242.1; OC43: YP_009555245.1; MERS: YP_009047211.1; SARS COV1:NP_828858.1, SARS-COV2: YP_009724397.2
