# Supplementary material for: Immunoinformatic Analysis of SARS-CoV-2 Nucleocapsid Protein and Identification of COVID-19 Vaccine Targets
Source: Front Immunol. 2020 Oct 28;11:587615. doi: 10.3389/fimmu.2020.587615 (PMC7655779; doi:10.3389/fimmu.2020.587615)
Supplement: Supplementary file 2 [file DataSheet_2.pdf]

Supplementary Table 1. Comparison of conserved T cell exposed motifs across the human coronaviruses

Sars-COV-2 Position	T cell exposed motif	peptide	229E	NL63	HKU1	OC43	MERS	SARS-COV1	SARS-COV2	Pocket positions
**MHC I – T cell exposed motifs ~~~4,5,6,7,8~**										
67	~~~QGVPI~	SDGQGVPIA			+					SDG~~~~~A
		VEGQGVPIA				+				VEG~~~~~A
		PRGQGVPIN						+	+	PRG~~~~~N
106	~~~YFYYL~	PRWYFYYLG			+	+		+	+	PRW~~~~~G
107	~~~FYYLG~	KLHFYYLGT	+	+						KLH~~~~~T
		RWYFYYLGT			+	+		+	+	RWY~~~~~T
108	~~~YYLGT~	LHFYYLGTG	+							LHF~~~~~G
		VHFYYLGTG		+						VHF~~~~~G
		WYFYYLGTG			+	+		+	+	WYF~~~~~G
109	~~~YLGTG~	HFYYLGTGP	+	+						HFY~~~~~P
		YFYYLGTGP			+	+		+	+	YFY~~~~~P
110	~~~LGTGP~	FYYLGTGPH	+	+		+				FYY~~~~~H
		FYYLGTGPY			+					FYY~~~~~Y
		FYYLGTGPE						+	+	FYY~~~~~E

254	~~~KPRQK~	ILTKPRQKR			+					ILT~~~~~R
		ILNKPRQKR				+				ILN~~~~~R
		ASKKPRQKR						+	+	ASK~~~~~R
255	~~~PRQKR~	LTKPRQKRT			+					LTK~~~~~T
		LNKPRQKRS				+				LNK~~~~~S
		SKKPRQKRT						+	+	SKK~~~~~T
**MHC II – T cell exposed motifs ~2,3~ 5~7,8~**										
102	PR~Y~YY	KQLLPRWYFYYLGTG			+					KQLL~~W~F~~LGTG
		RQLLPRWYFYYLGTG				+				RQLL~~W~F~~LGTG
		KQLAPRWYFYYTGTG					+			KQLA~~W~F~~TGTG
		KELSPRWYFYYLGTG						+		KELS~~W~F~~LGTG
		KDLSPRWYFYYLGTG							+	KDLS~~W~F~~LGTG
105	YF~Y~GT	LPRWYFYYLGTGPYA			+					LPRW~~Y~L~~GPYA
		LPRWYFYYLGTGPHA				+				LPRW~~Y~L~~GPHA
		APRWYFYYTGTGPEA					+			APRW~~Y~T~~GPEA
		SPRWYFYYLGTGPEA						+	+	SPRW~~Y~L~~GPEA
106	FY~L~TG	PKLHFYYLGTGPHKD	+							PKLH~~Y~G~~PHKD
		PKVHFYYLGTGPHKD		+						PKVH~~Y~G~~PHKD
		PRWYFYYLGTGPYAN			+					PRWY~~Y~G~~PYAN
		PRWYFYYLGTGPHAK				+				PRWY~~Y~G~~PHAK

		PRWYFYYLGTGPEAG						+		PRWY~~Y~G~~PEAG
		PRWYFYYLGTGPEAS							+	PRWY~~Y~G~~PEAS
107	YY~G~GP	KLHFYYLGTGPHKDA	+							KLHF~~L~T~~HKDA
		KVHFYYLGTGPHKDL		+						KVHF~~L~T~~HKDL
		RWYFYYLGTGPYANA			+					RWYF~~L~T~~YANA
		RWYFYYLGTGPHAKD				+				RWYF~~L~T~~HAKD
		RWYFYYTGTGPEAAL					+			RWYF~~T~T~~EAAL
		RWYFYYLGTGPEAGL						+		RWYF~~L~T~~EAGL
		RWYFYYLGTGPEASL							+	RWYF~~L~T~~EASL
**MHC II – T cell exposed motifs -1~~,3~ 5~7,8~**										
64	F~~G~G~PI	FKFSDGQGVPIAFGV			+					FK~SD~Q~V~AFGV
		FEFVEGQGVPIAPGV								FE~VE~Q~V~APGV
		LRFPRGQGVPINTNS								LR~PR~Q~V~NTNS
		LKFPRGQGVPINTNS								LK~PR~Q~V~NTNS
104	P~~Y~Y~LG	LLPRWYFYYLGTGPY			+					LL~RW~F~Y~TGPY
		LLPRWYFYYLGTGPH								LL~RW~F~Y~TGPH
		LSPRWYFYYLGTGPE								LS~RW~F~Y~TGPE
106	W~~Y~L~TG	PRWYFYYLGTGPYAN			+					PR~YF~Y~G~~PYAN
		PRWYFYYLGTGPHAK								PR~YF~Y~G~~PHAK
		PRWYFYYLGTGPEAS								PR~YF~Y~G~~PEAS

		PRWYFYYLGTGPEAG							PR~YF~Y~G~~PEAG
165	L~~G~Y~EG	TILPQGYYVEGSGRS			+				TI~PQ~Y~V~SGRS
		TVLPQGYYIEGSGRS							TV~PQ~Y~I~SGRS
		TTLPKGFYAEGSRGG							TT~PK~F~A~SRGG
